# Reports of Cases in Dr. Hamilton’s Surgical Dispensary, in the Medical Department of the University of Buffalo, with Remarks

**Published:** 1848-05

**Authors:** 


ART. VII.—*Reports of cases in Dr. Hamilton's Surgical Dispensary, in the Medical Department of the University of Buffalo, with remarks.*

*Case* 1.—Saturday, Feb. 26th. *Gunshot wound*; Shongo, an Indian, and grandson of the celebrated Mrs. Jemison, is deaf and dumb, and has only communicated by signs the manner in which the accident occurred. He was priming a pistol loaded only with powder, when the pistol was suddenly discharged, and its contents lodged in the palm of the left hand. "I saw him shortly after the accident, and found that the palmar fascia, the muscles and tendons were extensively torn, the lacerations extending between the carpal bones nearly through the hand, and widely under the skin in several directions. I can have no doubt but that the superficial and deep seated palmar arch were both lacerated, yet the hæmorrhage was very slight."

"The wound has been washed with soap and water, dressed with simple cerate alone, and as you see, it has been gradually and steadily granulating, and cicatrizing. This is the ordinary course of wounds when left to themselves. Simply keep a sore of this kind clean, and in nine cases out of ten, it will be restored more rapidly than under the use of all the ointments and washes and pain extractors and 'all healing salves' in the world."

"In this case it has been especially necessary to keep the fingers, extended with a splint to prevent contractions of the tendons and of the palmar fascia. For a description of the causes and phenomena of contractions of the palmar fascia, you may consult 'Dupuytren's Lectures." He was the first who properly distinguished these contractions from contractions of the tendons."

*Case* 2.—*Amputation of the fore and middle finger.* "This lad cut off his fingers about two weeks since in a straw cutting machine. I did not see him until eighteen hours after the accident. I then found it necessary to re-amputate for the purpose of obtaining proper flaps. The fore finger I amputated at the second articulation. The middle finger I amputated through the center of the second phalanx making the section of the bone with Liston's bone cutters. Both fingers are now healing kindly. It is dressed with simple cerate."

Before dressing the fingers, as the lad had always previously made great complaint when the hand was dressed, Dr. Harvey administered to him chloroform in presence of the class. He made four or five inhalations and although his consciousness was not lost, he allownd the dressings to be removed and re-applied without complaint. His sensibility was evidently diminished.

*Case* 3.—*Sprain of the shoulder followed by numbness and paralysis of arm.* "This shoulder was injured some months since, and as often happens in such cases a soreness and lameness has existed ever since; and gradually the chronic inflammation has extended from the muscles and ligaments to the neuralemes, and at length he has at first a pain shooting down to his elbow, next to his hand and fingers, and then a numbness of some part of the limb; here, it is along the parts chiefly supplied by the ulnar nerve. This is a singular fact, that pain and numbness are generally associated in paralyzed limbs, yet it is what every surgeon must have often noticed."

"Such serious results from apparently trivial injuries about the shoulder-joint are not uncommon. Sometimes it is induced by a rheumatic condition of the shoulder, sometimes by a blow or a sprain or a dislocation. In all cases it is a deep seated, chronic tendinous inflammation at first, and gradually at last involving the nerves of the axillary plexus."

"The treatment which I shall recommend, will be a succession of blisters on the shoulder, and daily frictions with a stimulating liniment, upon the arm and hand."

*Case* 4.—Wednesday, March 2d, 1848. *Fractured Ulna.* Mrs. ———, aged about 25, fell upon a log and struck upon the ulna about three inches above its lower extremity. This occurred several days since, but she has not presented herself for treatment until to-day.

"You notice the slight depression at the seat of fracture, the motion, and the prone condition of the hand: it is with great difficulty she can supine it."

The arm was dressed with a graduated compress laid along the palmar and dorsal surface of the forearm, especial care being taken to fill up the depression immediately above the ball of the thumb and little finger. Two broad splints were applied, one upon the dorsal and one upon the palmar surface, and after these was turned a roller. Dr. H. omitted the roller usually applied from the hand to the wrist, remarking that as the object was solely to prevent œdema, it could be of no service above the wrist, and that it might often do serious harm by pressing the broken bones against each other: and this might happen too when the roller was not applied at first unduly tight, since the subsequent swelling might be such as to tighten the roller injuriously at the seat of fracture. Omitting this first roller also we avoid all dangers of that serious accident, a loss of the arm from sloughing, which has so frequently happened in the practice of the best surgeons. The circulation of of the arm along its radial and ulnar sides is thus left wholly uninterrupted.

*Case* 5.—*Fractured Ulna.* Mrs. E. Smith, aged about 26. "Mrs. S. fell yesterday from a loaded wagon and broke the ulna of the right arm at the same point as the other patient, indeed, this is the point at which this bone is most frequently fractured. The hand is also, as in the other case, forcibly proned."

The same dressings were applied, and the arm laid in a sling against the side of the body, in a position *midway between supination and pronation*, for when the forearm lies across the chest the two bones are not parallel unless this position is assumed. The effect of supination or pronation in displacing the fragments was demonstrated on the skeleton.

*Case* 6.—*Labium Leporinum.* ——— ———, aged 11 years. This was operated upon when he was seven weeks old, but it was unsuccessful. The fissure is single and on the left side.

The operation was made in the usual mode except that Dr. H. used the common interrupted sutures instead of the hare-lip pins Dr. H. stated that he had operated a large number of times and alwavs used the common suture, and that he never had but one failure ; and in this case he had made the operation and then left the patient in charge of another physician, who not being accustomed to the mode of dressing, allowed the lip to stretch and tear open. "Hare-lip pins possess not a single advantage over the common suture, while they are liable to several objections, not the least of which is the difficulty of applying adhesive straps over them in such a manner as to support the lips in contact, without permitting any strain to fall upon the sutures."

At the end of forty-eight hours the sutures were removed, and the adhesive straps were finally removed on the 10th day. The cure was complete.

*Case 7.* — *Labium Leporinum.* Child aged seven weeks. This child had a very wide fissure, connected with the alveoli, hard and soft palate. It could not nurse and was with difficulty fed. The danger of operating at this age was stated both to the class and to the parents. The danger is that convulsions may result; one case was mentioned in which this had happened and the patient died before the operation was completed.

The operation was made in the same manner as in the first case. It was completed very quickly, yet the child became very pale, but after it was carried out it was heard to cry lustily, and it was presumed to be safe. W. W.

